# Investigations into Ti-15Mo-W Alloys Developed for Medical Applications

**DOI:** 10.3390/ma12010147

**Published:** 2019-01-04

**Authors:** Mihai Buzatu, Victor Geantă, Radu Ştefănoiu, Mihai Buţu, Mircea-Ionuţ Petrescu, Mihai Buzatu, Iulian Antoniac, Gheorghe Iacob, Florentina Niculescu, Ştefan-Ioan Ghica, Horaţiu Moldovan

**Affiliations:** 1 MedLife-Medical Center, 222 Calea Victoriei, 031291 Bucharest, Romania; buzatu.mihai@yahoo.com; 2 Faculty of Materials Science and Engineering, University “Politehnica” of Bucharest, Spl. Independenţei 313, Sector 6, 060042 Bucharest, Romania; victor.geanta@upb.ro (V.G.); radu.stefanoiu@upb.ro (R.Ş.); mihaibutu@yahoo.com (M.B.); ipetrescu@yahoo.com (M.-.I.P.); mbuzaturo@yahoo.com (M.B.); antoniac.iulian@gmail.com (I.A.); flori.pereteanu@yahoo.com (F.N.); 3 UMF Carol Davila, 37 Dionisie Lupu, 020021 Bucharest, Romania; ghica_stefan@yahoo.com; 4 Faculty of Medicine, Titu Maiorescu University, 22 Dambovnicului Str, 040441 Bucharest, Romania

**Keywords:** metallic biomaterials, titanium, re-melting, hardness, stress–strain

## Abstract

The β-Ti alloys have attracted the attention of researchers due to their excellent properties and their remarkable biocompatibility. The present study evaluated the mechanical behavior analysis (hardness, compressive strength, and modulus of elasticity) of the Ti-15Mo-W system. For experimental research, we chose the TiMo15 biocompatible alloy as a starting material. In order to improve the mechanical properties, we added tungsten amounts of 3.88 to 12.20 wt.% and analyzed the results obtained. The successive melting of the samples was done using a vacuum arc furnace in a copper crucible cooled with water. Following micro-structural investigations, we found this alloy possessed a homogeneous structure and showed β-phase predominance. The investigated alloys have good mechanical properties—the mean Vickers micro-hardness values are between 251 to 321 HV, the compressive strength values range from 717 to 921 MPa, and the modulus of elasticity is between 17.86 and 45.35 GPa. These results are compatible to the requirements of a metallic material for medical applications as artificial implant devices.

## 1. Introduction

A commonly encountered problem in the older population and in other types of pathology is the issue of impaired mobility. The replacement of the affected organs has been addressed by the use of various types of metallic implants (plates, joint prostheses, spinal fixation devices). So far, these were the best available materials to sustain the high pressures and high forces applied to them [1]. The replacement biomaterials require a low modulus of elasticity, as close as possible to that of bone (10 to 30 GPa), to prevent bone resorption and promote adequate remodeling [2].

From the plethora of various metallic materials, only certain types can be successfully used as biocompatible implants [3]. Biomaterials are natural, synthetic, or composite structures, and when in contact with living tissues, they seldom affect their biological properties and they can even enhance their parameters [4].

Metallic implants were primarily developed for fixing bone fractures of various causes. Irrespective of their primary source (iron, silver, or gold), almost all attempts to use them were unsuccessful until the implementation in 1860 of Lister’s aseptic surgical technique [5].

Since then, metallic materials have had a major role in the design of various orthopedic devices such as bone plates, screws and nails, and permanent implants (e.g., hip replacement prostheses) [6]. Various types of alloys found many applications in the dental practice as well [7]. Recently, research efforts in the domain of metallic biomaterials led to the use of shape memory alloys in reconstructive surgery of various tissue/structures. Ni-Ti combinations are now widely used for vascular stents [8], and Mg-based alloys are successfully applied for bone regeneration and remodeling [9].

Biocompatible materials with long-lasting applications are very few, even though there are a multitude of industrial alloys [10,11,12,13]. Of the metallic materials for this purpose, Ti and Ti-based alloys account for over 40%.

Lately, Ti-based alloys are intensively investigated for various applications. Their special properties (super-plasticity, shape memory effect, low modulus of elasticity, bio-corrosion resistance, remarkable biocompatibility) [14] brought upon the exciting field of implant research for various environments, modern bone fracture therapies being a leading example [15,16].

Due to various medical applications, the expected properties of these biocompatible materials are required to be of a very high standard and to be used only under strict conditions. Alloys can release various types of metallic ions, and some of them have adverse effects that can even promote medical implant rejection. Therefore, a metallic implant must be non-toxic to the human body and thus not cause allergenic or inflammatory reactions [17]. However, a metallic material used with a high success rate as an implant in orthopedics might not be suitable in the vascular field due to its thrombogenic properties. It is very important to know the general and specific properties of the various metallic materials. These features must be anticipated before designing any medical application, but these must also be considered in relation to the changes that may occur over time while implanted [18,19]. A universally valid definition provided by the U.S. Food and Drugs Administration [20] states that the material should not induce any measurable harm to the host.

Titanium is a non-toxic biocompatible material irrespective of the method of penetration (dermal, inhalation, ingestion), even in high doses [21]. It is estimated that 0.8 mg of titanium is ingested by humans every day, but most of it is eliminated without being absorbed [22]. Titanium implants are easily accepted into the human body and are physically well integrated into the bone tissue. Due to the non-magnetic properties, the patients with titanium implants can safely be subjected to magnetic resonance investigations. In vitro, titanium may still cause genetic changes in connective tissue [23,24]. In vivo, titanium particles with specific dimensions can biologically affect white blood cells [25].

As implant metallic biomaterials, Ti-based alloys are used intensively because of the very good biological compatibility, excellent mechanical properties, and their corrosion-resistance to the fluids of the human body and the environment. Recently, titanium β-alloys become one of the most current subjects in the domain of biomedical Ti alloys for the lower elastic modulus compared to the classical titanium alloys [26,27,28,29]. Phase stability and some mechanical properties and corrosion properties in Ti-based alloys can be predicted by parameters such as the order of the bond (Bo), the energy level of the orbital d (Md) [30], the valence electron/atom ratio (e/a) [5], and the equivalent of Mo [31].

The main objective of the study was to obtain a material with the elasticity modulus as close as possible to that of a human bone. Choosing tungsten as part of the alloy improved the parameters Md Bo and e/a and consequently achieved characteristics near to its values [1,2].

Comparing the stainless steels and Co alloys to the Ti-based alloys demonstrated the Ti-based alloys to be superior in biocompatibility, mainly due to their excellent corrosion resistance [19,29]. The Ti-6Al-4V (Ti64) alloys, which represent the first generation of Ti-based bio-alloys, were reported to cause many allergic reactions [32]. Next, the research was channeled towards the Ti β-alloys, which represent the second generation of Ti-based bio-alloys. Some β-stabilizing elements, such as Mo, Nb, Ta, and Zr, are used as alloying elements. These are considered to be relatively safe compared to V and Al [33]. So far, there are not many long-term clinical tests on the biocompatibility of Ti β-alloys.

Biocompatibility and resistance to corrosion are key elements for a successful application of the metallic implants in the clinical practice.

## 2. Experimental Procedures

Due to the very good biocompatibility of tungsten [34], we used it together with the well-known Ti-15Mo alloy to develop a new Ti-15Mo-W biomaterial where the tungsten content ranged from 3.88 to 12.20 wt.%, thus obtaining mechanical properties fit for the purpose of implant manufacturing suitable in the medical field.

To prepare the alloy, the double or triple re-melting method was done using a vacuum arc remelting equipment (VAR) model MRF ABJ-900 (Materials Research Furnaces, Inc., Suncook, NH, USA). Multiple melting was used due to the significant differences in densities and melting temperatures between titanium and molybdenum, which can create unwanted segregation problems.

**Figure 1:**
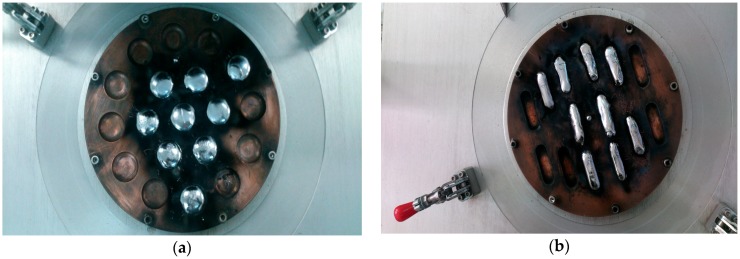
Images of the specimens obtained after re-melting in a vacuum arc furnace: (**a**) tablet; (**b**) bars.

Ti-15Mo with different tungsten contents (tungsten was added to reduce the modulus of elasticity) were melted using a vacuum arc furnace in a copper crucible cooled with water. Two types of samples were obtained (Figure 1)—circular tablets of 30 mm in diameter for structural and microstructural analysis and bars of 60–150 mm long for mechanical tests.

The micro-structural analyses were performed using a scanning electron microscope Quanta Inspect F50 (Thermo Fisher Scientific Inc., Waltham, MA, USA) with a field emission gun (FEG, Thermo Fisher Scientific Inc., Waltham, MA, USA) with 1.2 nm resolution and an X-ray diffractometer PANalytical X’Pert PRO MRD model (PANalytical, Malvern, UK) with the wave length of l_Cu_ = 1.544.

The hardness was measured using a Vickers micro-hardness tester (Shimadzu Research Laboratory (Europe) Ltd., Manchester, UK) at a load HV0.2 of 1961 N and a duration of 15 s. The Vickers hardness determination was made of two alloys, Ti15Mo9W and Ti15Mo5W, and an average of 10 tests were performed. The tests started from the center of the sample to the exterior and results were obtained by averaging these values. The sample had a diameter of 15 mm and a height of 20 mm.

The compression tests were done using the universal machine for mechanical tests EDZ 40 (Capacity: 400 kN/40,985 KgF, WMW Heckert, Leipzig, Germany). For compression tests and the determination of the modulus of elasticity, from the bars obtained after melting (Figure 1b), the cylindrical samples with the diameter of the base 10 mm and the height of 15 mm were cut by turning.

## 3. Results and Discussion

The micro-structural examination of the alloys obtained in the casting state was aimed at observing the compactness and homogeneity of the alloy structure, the morphology of the grains, the morphology and the distribution of the component phases, and the distribution of the alloying elements.

The use of molybdenum in the form of master alloy instead of elemental Mo additions may overcome the tendency of Ti-Mo segregation from the primary melting. They can also be used to create a homogeneous alloy composition and other melting techniques, such as electron melting using a shallow melt bath, yet such processes do not solve the problem of segregation in one melting. After re-melting, the alloys with the chemical composition listed in Table 1 were obtained.

The investigations led to the existence of two groups of alloys: alloys with low tungsten content—Ti15Mo (4 to 6) W, biphasic beta alloys with small amounts of hexagonal omega phase, and alloys with higher tungsten content—Ti15Mo (7 to 11) W, monophasic beta alloys.

**Figure 2:**
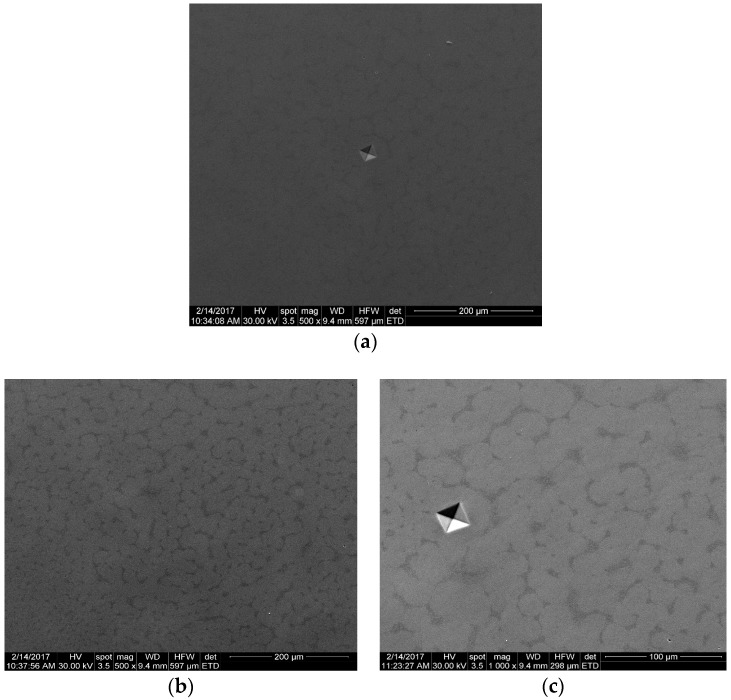
Scanning electron microscope images of as-cast Ti-15Mo-W alloys: (**a**) Ti15Mo5W; (**b**) Ti15Mo7W; (**c**) Ti15Mo9W.

**Figure 3:**
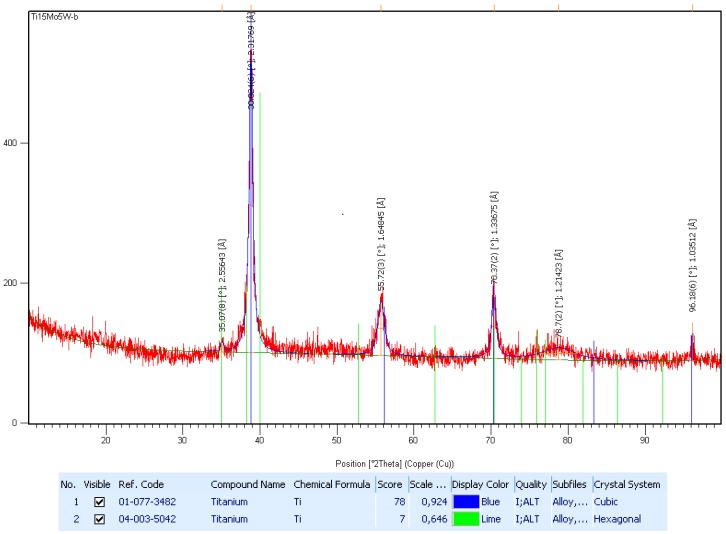
The X-ray diffractometer pattern for Ti15Mo5W alloy; the peaks are corresponding to β- and ω-phases.

In Figure 2, the SEM micrographs of the studied alloys are presented. A homogeneous microstructure and specific Vickers micro-hardness indentation marks were observed. The micro-structural characterization of the alloys proved to be homogeneous; the developed alloys had β-phase predominance (Figure 2 and Figure 3).

In Figure 3, the X-ray analysis of the Ti15Mo5W alloy is shown on a diffraction range of 2θ = 30–90°. This was indexed in order to identify the structural phases, and the diffraction spectrum was found to have maxima corresponding to the beta and omega phases. The distribution of the ω-phase (hexagonal system) within the β-phase (cubic system) regions is relatively low. Thus, we can assume that the alloy can be classified as having a near-monophasic structure (see supplementary Table S1).

**Figure 4:**
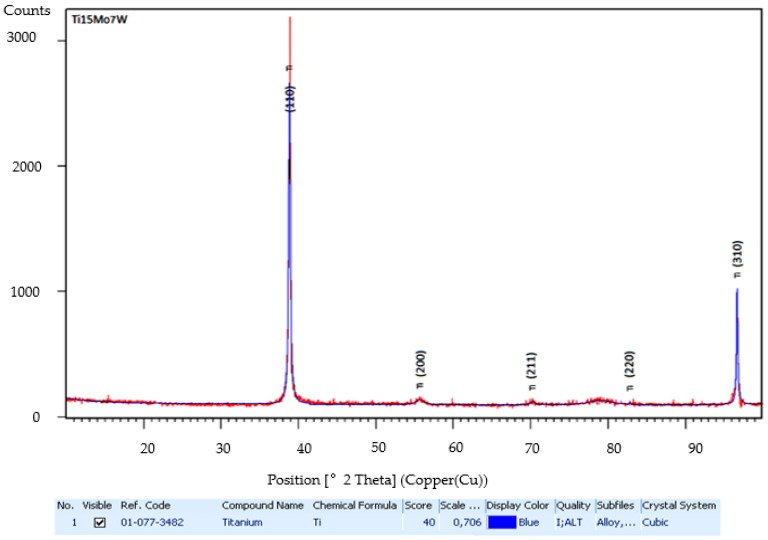
The X-ray diffractometer pattern for Ti-15Mo-7W alloy; the peaks are corresponding to β-phase.

In Figure 4, the X-ray analysis of the Ti15Mo7W alloy is shown on a diffraction range of 2θ = 20–100°. The diffraction spectrum was found to have maxima corresponding to the β-phase (see supplementary Table S2).

Mechanical behavior analysis (hardness, compressive strength, and modulus of elasticity) aimed to determine some mechanical properties of these newly obtained alloys in order to fulfill the mechanical bio-functioning conditions necessary for use as medical materials—mainly a low modulus of elasticity.

**Figure 5:**
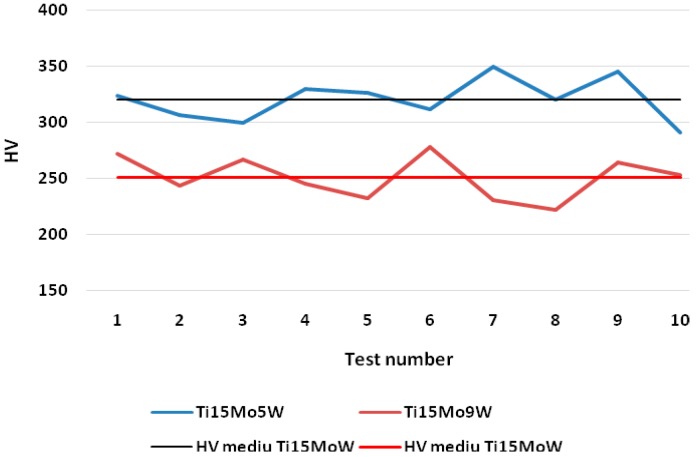
Comparative results of the micro-hardness values for Ti15Mo5W and Ti15Mo9W alloys and their average values.

According to the micro-structural analyses, the existence of two groups of alloys was counted, namely alloys with low tungsten content (Ti15Mo [4 to 6] W) and alloys with higher tungsten content (Ti15Mo [7 to 11] W). For the micro-hardness analysis, one representative alloy of each group was chosen. The test results of Vickers micro-hardness for the two alloys are presented in Figure 5. Micro-hardness values ranging from 300–350 HV with an average of 321 HV for the Ti15Mo5W alloy and values ranging from 222–278 HV with an average of 251 HV for the Ti15Mo9W alloy were obtained. The small amounts of ω-phase in the structure of the Ti15Mo5W alloy, which crystallized in the hexagonal system, explain the slightly higher hardness of this alloy.

The values of the compression tests and modulus of elasticity are presented in Table 2.

Compressive strength and modulus of elasticity are also influenced by the existence of phases in the structure of the two groups of alloys. The stability of the cubic structure in the second group of alloys (monophasic beta alloys) has an influence on the modulus of elasticity. The difference between the values of the mechanical properties is given by the influence of the material parameters: bond order (Bo), the energy level of metal d-orbital (Md), the ratio of valence electrons/atom (e/a), and the equivalent of Mo (Mo_eq_), as demonstrated in previous studies [26,35].

The additions of W grow the values of the e/a ratio and the equivalent of Mo. Thus, the phase stability of the alloy Ti-15Mo increases by the addition of W, which allows a biocompatible material with a low modulus of elasticity to be obtained under the increase of mechanical strength.

**Figure 6:**
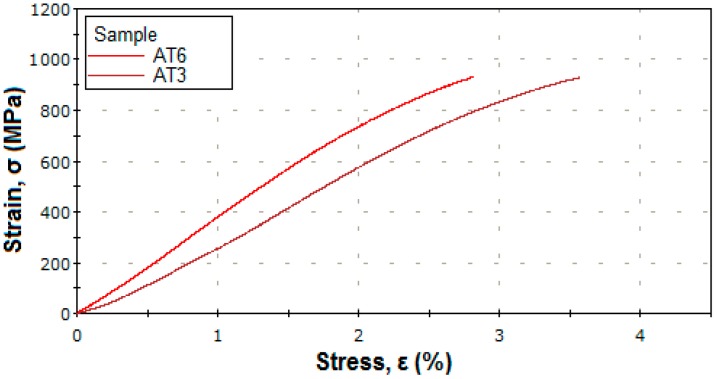
Tensile stress–strain curves of the sample 3 (Ti15Mo11W) and sample 6 (Ti15Mo10W) to a force applied by 95 kN.

**Figure 7:**
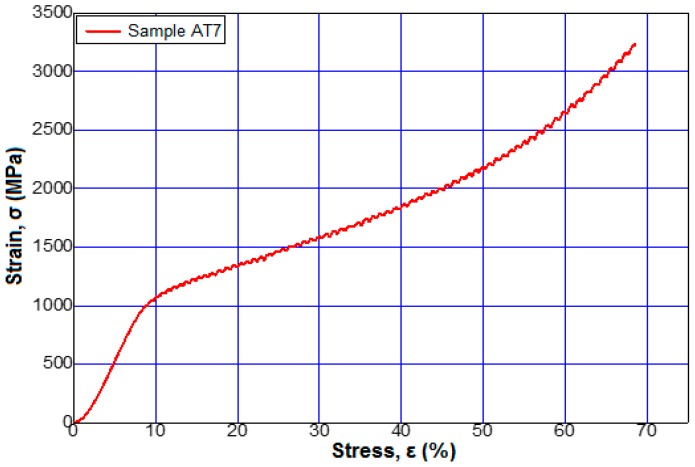
Tensile stress–strain curves of the sample 7 (Ti15Mo9W) to a force applied by 250 kN.

**Figure 8:**
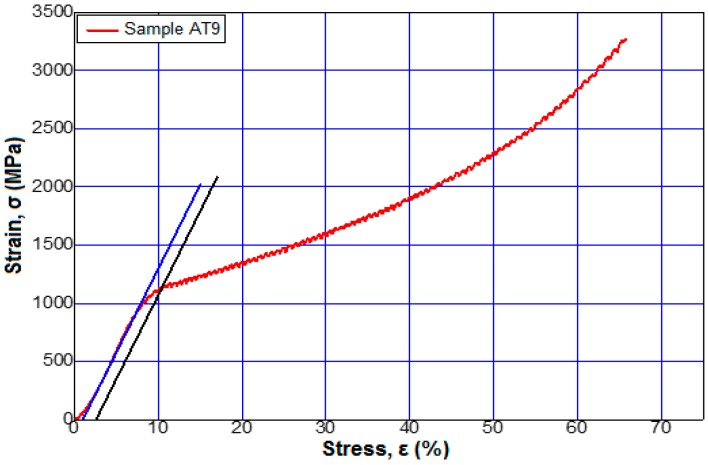
Tensile stress–strain curves of the sample 12 (Ti15Mo8W) to a force applied by 250 kN.

**Figure 9:**
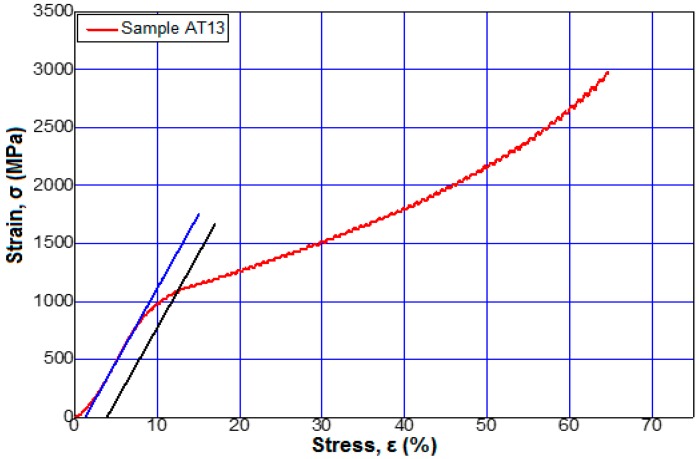
Tensile stress–strain curves of the sample 13 (Ti15Mo7W) to a force applied by 250 kN.

Figure 6 shows the stress–strain curves for alloys 3 and 6 from Table 2 at a maximum applied force of 95 kN. Figure 7, Figure 8 and Figure 9 show the stress–strain curves for alloys 7, 9, 12, and 13 from Table 2 at a maximum applied force of 250 kN.

The stress–strain curves of all the alloys obtained, whose chemical compositions are presented in Table 1, had the same behavior.

Regarding the influence of the tungsten content on the elasticity modulus values of the studied alloys, it was observed that minimal values were obtained near the modulus of elasticity of the bone, (about 20 GPa) [1,2] for Ti15MoW alloys samples AT10 to AT13.

## 4. Conclusions

The investigations demonstrated the achievement of a homogeneous metallic material in terms of the structure and chemical composition. The alloy microstructure was compact and homogeneous and was formed from relatively equidistant polyhedral grains. The results demonstrated that the alloys with a higher content of tungsten had a lower hardness. The alloys with a higher content of tungsten had greater β-phase stability, and the alloys with a smaller content of tungsten contained small amounts of ω-phase (hexagonal crystallization system). After the deformation at a maximum force of 40 tf, the samples showed no cracks, indicating super-plasticity characteristics as well as other low elasticity titanium alloys. The investigated alloys have good mechanical properties, especially the modulus of elasticity, which are appropriate to the requirements of a metallic material for medical applications.

## Figures and Tables

**Table 1:** Chemical compositions of the alloys from Ti-15Mo-W system.

Element wt.%	Alloy														
	AT1	AT2	AT3	AT4	AT5	AT6	AT7	AT8	AT9	AT10	AT11	AT12	AT13	AT14	AT15
Ti	78.58	80.35	75.14	78.05	76.37	79.61	75.64	76.30	76.00	77.74	80.25	77.77	78.01	80.58	71.10
Mo	15.67	15.55	13.78	15.90	15.41	13.28	15.15	15.02	15.82	14.97	15.51	14.22	15.11	15.54	16.07
W	5.75	4.10	11.08	6.05	8.22	9.45	9.21	8.68	8.18	7.29	4.24	8.01	6.88	3.88	12.20

**Table 2:** Results of compression tests.

Mechanical Test	Alloy														
	AT1	AT2	AT3	AT4	AT5	AT6	AT7	AT8	AT9	AT10	AT11	AT12	AT13	AT14	AT15
**R_c_ [MPa]**	801	717	815	901	808	916	801	921	791	908	782	821	912	798	798
**E [GPa]**	44.23	42.78	33.51	43.14	45.35	35.22	32.19	36.36	31.11	23.27	22.48	17.86	19.55	43.05	43.32

## Supplementary Materials

The following are available online at http://www.mdpi.com/1996-1944/12/1/147/s1, Table S1: The X-ray diffraction indexation sheet in Figure 3, Table S2: The X-ray diffraction indexation sheet in Figure 4.
