# Supplementary material for: Investigations into Ti-15Mo-W Alloys Developed for Medical Applications
Source: Materials (Basel). 2019 Jan 4;12(1):147. doi: 10.3390/ma12010147 (PMC6337162; doi:10.3390/ma12010147)
Supplement: Supplementary file 1 [file materials-12-00147-s001.pdf]

Supplementary Material for: Investigations into Ti-15Mo-W Alloys Developed for Medical Applications

Mihai Buzatu, Victor Geantă, Radu Ştefănoiu, Mihai Buţu, Mircea-Ionuţ Petrescu, Mihai Buzatu, Iulian Antoniac, Gheorghe Iacob, Florentina Niculescu, Ştefan-Ioan Ghica and Horaţiu Moldovan

Table 1. The X-ray diffraction indexation sheet in Figure 3.

**Peak list**								
**No.**	h	k	l	d [A]		2Theta (deg)		I (%)
**1**	1	1	0	2,31250		38,914		100,0
2	1	0	1	2,30700		39,011		100,0
3	2	0	1	1,63150		51,088		8,3
4	2	0	1	1,63200		56,328		75,0
5	0	0	2	1,40650		66,415		4,3
6	3	0	0	1,33900		70,238		75,0
7	2	1	1	1,33310		70,596		18,7
8	1	1	2	1,20400		79,551		80,0
9	2	2	0	1,16200		83,044		5,0
10	2	2	1	1,06940		92,160		0,8
11	3	1	1	1,03320		96,412		5,8
12	4	0	0	1,00130		100,582		0,1
**Structure**								
No.	Name	Elem.	X	Y	Z	Biso	sof	Wyck.
1	TI1	Ti	0,0000	0,0000	0,0000	0,5000	1,0000	1a
2	TI2	Ti	0,3333	0,6666	0,5000	0,5000	1,0000	2d

Table 2. The X-ray diffraction indexation sheet in Figure 4.

**Peak list**								
**No.**	h	k	l	d [A]		2Theta (deg)		I (%)
**1**	1	1	0	2,31650		38,845		100,0
2	2	0	0	1,63800		56,103		13,2
3	2	1	1	1,33740		70,335		20,9
4	2	2	0	1,15820		83,377		5,4
5	3	1	0	1,03600		96,066		6,7
6	2	2	2	0,94570		109,082		1,6
7	3	2	1	0,87550		123,246		8,3
8	4	0	0	0,81900		140,284		0,9
**Structure**								
No.	Name	Elem.	X	Y	Z	Biso	sof	Wyck.
1	TI1	Ti	0,0000	0,0000	0,0000	0,5000	1,0000	2a

© 2019 by the authors. Licensee MDPI, Basel, Switzerland. This article is an open access article distributed under the terms and conditions of the Creative Commons Attribution (CC BY) license (http://creativecommons.org/licenses/by/4.0/).
